# Quarantining From Professional Identity: How Did COVID-19 Impact Professional Identity Formation in Undergraduate Medical Education?

**DOI:** 10.5334/pme.1308

**Published:** 2024-02-20

**Authors:** Maham Rehman, Faran Khalid, Urmi Sheth, Lulwa Al-Duaij, Justin Chow, Arden Azim, Nicole Last, Sarah Blissett, Matthew Sibbald

**Affiliations:** 1 Michael G. Degroote School of Medicine, CA; 2 Department of Family Medicine, University of Ottawa, CA; 3 Department of Medicine, Division of Cardiology, McMaster University, CA; 4 Department of Medicine, Division of General Internal Medicine, McMaster University, CA; 5 Centre for Simulation Based Learning, McMaster University, CA; 6 Department of Medicine, Division of Cardiology, Western University, CA

## Abstract

**Introduction:**

Professional Identity Formation (PIF) entails the integration of a profession’s core values and beliefs with an individual’s existing identity and values. Within undergraduate medical education (UGME), the cultivation of PIF is a key objective. The COVID-19 pandemic brought about substantial sociocultural challenges to UGME. Existing explorations into the repercussions of COVID-19 on PIF in UGME have predominantly adopted an individualistic approach. We sought to examine how the COVID-19 pandemic influenced PIF in UGME from a sociocultural perspective. This study aims to provide valuable insights for effectively nurturing PIF in future disruptive scenarios.

**Methods:**

Semi structured interviews were conducted with medical students from the graduating class of 2022 (n = 7) and class of 2023 (n = 13) on their medical education experiences during the pandemic and its impact on their PIF. We used the Transformation in Medical Education (TIME) framework to develop the interview guide. Direct content analysis was used for data analysis.

**Results:**

The COVID-19 pandemic significantly impacted the UGME experience, causing disruptions such as an abrupt shift to online learning, increased social isolation, and limited in-person opportunities. Medical students felt disconnected from peers, educators, and the clinical setting. In the clerkship stage, students recognized knowledge gaps, producing a “late blooming” effect. There was increased awareness for self-care and burnout prevention.

**Discussion:**

Our study suggests that pandemic disruptors delayed PIF owing largely to slower acquisition of skills/knowledge and impaired socialization with the medical community. This highlights the crucial role of sociocultural experiences in developing PIF in UGME. PIF is a dynamic and adaptable process that was preserved during the COVID-19 pandemic.

## Introduction

Professional identity formation (PIF) is a dynamic process whereby individuals acquire and internalize the values and beliefs of a particular profession [1]. The literature presents two complementary perspectives for understanding PIF: the individualistic lens and the sociocultural lens [2,3]. The individualistic lens emphasizes the significance of cognition, personal attributes, and social categories in shaping PIF [2]. From this orientation, PIF is the process where an individual strives to comprehend their own identity in relation to their personal attributes, aligning these traits with the desired values and attitudes of a physician. The sociocultural lens firmly situates an individual’s PIF within the broader influence of the social environment. This recognizes that one’s identity development is intricately intertwined with their social context and that identities take shape through social interactions, relationships, and contextual influences [2,3,4]. These perspectives collectively explore how our sense of self interacts with our environment to shape our professional identity [5]. In the context of undergraduate medical education (UGME), PIF encompasses the transformative journey of medical students evolving into competent healthcare providers [1].The environment and context in which physicians-in-training operate plays a crucial role in PIF development [6]. The COVID-19 pandemic significantly disrupted this contextual landscape [7,8]. The consequences of the pandemic on medical students’ PIF, both in the short and long term, remain unclear. In an era characterized by constant environmental change, we sought to understand how the COVID-19 pandemic influenced PIF in UGME from a sociocultural perspective.

PIF in UGME is a dynamic and non-linear process involving the unlearning, relearning, and construction/deconstruction of identity. It entails reflection, reflexivity, and critical consciousness, alongside social experiences like patient-physician interactions and interprofessional collaboration [2,9]. UGME incorporates specific practices such as professional competency education, reflective writing, and objective structured clinical exams to foster the adoption of healthcare professional values, beliefs, and responsibilities [3,10]. The personal and professional aspects of identity intersect in a double helix, where the individual and the profession intertwine and mutually shape each other [11].

The literature presents multiple approaches to understanding PIF in UGME, examining through an individual or sociocultural lens, centering three key principles: (i) the significance of experience and exposure to the clinical environment, (ii) the navigation of relationships with oneself, colleagues, and the clinical environment, and (iii) professional socialization [12,13,14]. Preliminary literature examining PIF in the context of the COVID-19 pandemic has largely taken an individualistic approach with current scholarly work exploring its impact on medical students’ PIF. It delves into learners’ attitudes towards COVID-19, cultivation of attributes like resilience, and tension between personal and professional values [15,16,17]. However, discourse notably overlooks sociocultural influences such as relationship building, perception, and direct changes to the clinical setting. The COVID-19 pandemic brought about substantial sociocultural changes. Shifts to online classes, limited access to healthcare settings, and reduced social interaction due to lockdowns have impacted PIF within UGME creating a disruptive environment [4,14,18]. There are sociocultural frameworks that can be used to understand the impacts of COVID-19 on PIF in UGME. One such framework is the Transformation in Medical Education (TIME) framework which outlines six domains of PIF: attitudes, personal characteristics, duties and responsibilities, habits, relationships, and perception and recognition [19]. The TIME framework applies a sociocultural lens to PIF, highlighting the crucial role of our environment and social context in fostering professional development [19].

Enhancing our grasp of PIF from a sociocultural perspective illuminates the dynamics of PIF amidst disruptive environments. This understanding equips us to extend support to PIF in a post-pandemic era and take proactive steps in navigating future disruptive scenarios. Therefore, to gain a comprehensive understanding of the pandemic’s effect on PIF, our study aims to examine how sociocultural aspects of PIF were impacted in UGME.

## Methods

We conducted a theory-informed, qualitative content study using semi-structured interviews exploring medical students’ experience in the UGME processes during the COVID-19 pandemic.

### Setting

**Figure 1:**
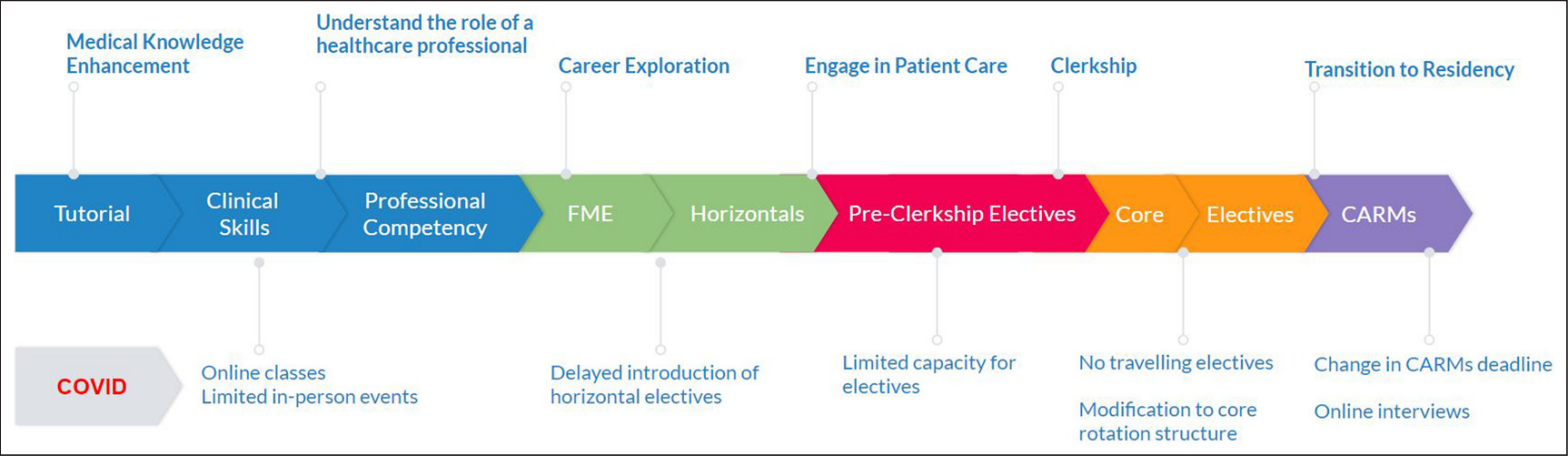
Process of professional identity formation and changes associated with the COVID-19 pandemic.

The study occurred at McMaster University, a mid-sized (200 students/year) Canadian medical school, with a three-year problem-based learning (PBL) program. PBL is a student-led pedagogy in which complex real world problems coupled with group based discussion drive learning [20]. There are limited large group sessions and didactic lectures. In pre-clerkship, students engage in self-directed learning, guided by tutors in tutorial groups. Group learning is supplemented by structured clinical skills sessions and a professional competency curriculum. Pre-clerkship includes career exploration through “horizontal electives”, which function as observerships to specialties. Students also complete three, two-week pre-clerkship electives in chosen specialties. In clerkship, students complete core rotations and choose five clinical electives in their preferred specialties. After these rotations, students apply for residency programs through the Canadian Resident Matching Service (CaRMS). The institution’s professional identity formation process is shown in Figure 1.

### Recruitment & Participants

We utilized convenience sampling, engaging participants with class-wide emails. Our study included the first 20 participants to volunteer – 9 from the Class of 2022 (c2022) and 11 from the Class of 2023 (c2023). These cohorts were chosen because the pandemic disrupted over 50% of their medical education. For c2022, it affected pre-clerkship and the beginning of clerkship; for c2023, it disrupted pre-clerkship and part of clerkship.

### Reflexivity

We formed a diverse research team, comprising healthcare professionals from various career stages. Team members include MS, an educational scholar and associate dean at McMaster University; SB, an educational scholar and program director at Western University; JC, a practicing cardiologist and clinician educator; AA, an internal medicine fellow and education scholar; FK, a University of Ottawa family medicine resident; MR and US, McMaster MD students experienced in qualitative research; LA, a critical care clinical fellow; and NL, a seasoned standardized patient trainer at McMaster’s Simulation-based Learning Center with extensive experience in qualitative methods and interviewing techniques. Within our research team, multiple members (MR, FK, US, JC, AA, LA) identify as people of color and have encountered racialization. MR and FK experienced undergraduate medical education during the COVID-19 pandemic, while the postgraduate medical education journeys of AA, LA, and JC were also affected by the pandemic. All team members shared perspectives on the importance of socio-cultural interactions in PIF development and on the possibility that COVID impacted PIF development. We were cognizant of the complexities of PIF and the personal hurdles posed by the pandemic.

### Data Collection

Twenty semi-structured interviews were conducted over Zoom between August 2022 to February 2023 by MR to maximize consistency and data quality. Given the interviewer was a medical student, the interviewer openly acknowledged any existing peer relationships with interviewees to maximize the quality of data obtained. All participants provided written, informed consent prior to interviews. Interviews were semi-structured, lasting 30–45 minutes. The research team developed the semi-structured interview guide and iteratively revised it during data collection. Interviews aimed to: 1) explore participants’ experiences in the medical program, 2) discuss crucial facets of their professional identity development, and 3) explore the extent of COVID-19’s impact on PIF (See Appendix A). The questions on how COVID impacted PIF explored all six domains of the TIME framework for PIF development [19]. Interviews were audio recorded, and transcribed via the Scribie software (www.scribie.com). Data collection was terminated when we agreed the analysis had adequate conceptual depth, was conceptually plausible, and was externally relevant to the broader academic community [21,22]. Each participant received a $10 CAD honorarium.

### Ethical Considerations

Ethics approval (2022–14219-GRA) was obtained from the Hamilton Integrated Research Ethics Board (HiREB).

### Data Analysis

A directed content analysis approach was selected as theoretical frameworks were being extended into a new context [23,24]. We took a deductive approach, with coding informed by the TIME framework. However, our analytic approach allowed for induction when themes did not align with our deductive codes. Our coding framework is presented in the Appendix B.

Six members of the research team (MR, US, JC, SB, MS, and LA) coded the transcripts. Aligning with an interactive approach, the coding team met regularly to review transcripts from interviews. We used Dedoose (*SocioCultural Research Consultants, LLC, Manhattan Beach, California)* as a data management tool. We took several steps to enhance rigor. We maintained a transparent audit trail – safeguarding raw data, transcripts, meeting minutes, and process notes. Interviewer reflections were documented as memos after each interview. Prior to and during coding and analysis, the research team engaged in reflexive practices. Our data analysis took a”dis-interested” perspective, and was attentive to identifying discrepant themes [25].

## Results

Based on our interview of 20 participants (See Table 1 for participant demographics) we uncovered five themes: disconnectedness, heroism, delayed transition, shifting paradigms, and influence on PIF. These themes were consistent with the domains of PIF outlined in the TIME framework. We also noticed the emergence of ‘disruptive environments’ which did not neatly align with the pre-existing domains of the TIME framework. As a result, we recognized its significance and incorporated it as a distinct and separate theme of ‘components of transformation’.

**Table 1:** Participant demographics.


ASPECT	NUMBER OF PARTICIPANTS

*Prior Education:*	

After undergraduate degree	16

After postgraduate degree	4

*Background:*	

Non-traditional	2

Nursing	2

Self-identified as people of color	15

First generation medical students	14

*Geographic Distribution:*	

Ontario (Total)	16

Greater Toronto Area	9

Other Canadian Regions	4


### Disconnectedness due to COVID-19

Unsurprisingly, the COVID-19 pandemic was socially isolating for many medical students. Due to repeated lockdowns and online learning, participants had limited in-person peer interaction. Platforms such as Zoom were imperfect in facilitating genuine connections. As a result, participants felt disconnected from their peers, educators, and the healthcare environment. They expressed a sense of awkwardness when interacting with members of their cohort and conveyed missing a sense of collegiality and comfort:

*“There was a lack of socialization at that time, we were just starting to kind of get comfortable and familiar with each other as a cohort and those kinds of links and that familiarity were definitely really disrupted to the extent that we were very uncomfortable around each other when we would see each other around in the hospital.”* (P19)

Due to classes being online, there was also a notable disconnect with educators. Students were unable to have informal conversations with their teachers after class, as one would in an in-person setting. This impacted students’ ability to network and find role models and mentors. One student expressed difficulty finding role models, especially when compared to the experiences of residents, who completed their medical education before the pandemic.

“*Tutors from their medical foundations [who] encouraged them to do horizontals with them or observerships with them. Our tutors were just simply not allowed to do that. Or we were not allowed to do that. So it was hard to find role models. It was really hard to develop mentor and mentee relationships.”* (P5)

The need for role models and mentors was especially important for students as they recognized that the connections formed in pre-clerkship had the ability to guide career choices and opportunities.*“I think being online and during COVID pandemic, we were restricted in relationships that we could form both with our colleagues and with the medical community, and I think medicine is a lot about relationships, it’s the first… Sometimes the relationship you form helps you choose a specialty versus the actual content of the specialty*.” (P1)

From a sociocultural lens the onset of virtual engagement for learning and connection produced social gaps between learners, peers and educators, ultimately creating a disconnect with the healthcare system. Participants remarked feeling disengaged with their role as a medical student. Some students felt a reason for this feeling was that they “*didn’t have to move from their parents’ house for at least the first few months of med school* (P4).” Others attributed it to the online environment explaining that “*it’s a lot easier to tune out (P7)*,” and “*who can pay attention to Zoom for that long? (P6)*.” One participant expressed “*losing purpose a little bit* (P12),” with the online environment. It was only when they entered the clerkship environment and engaged in in-person learning that they began to feel like a valuable part of the healthcare system. *“I saw myself as a medical student in pre-clerkship and now during clerkship I can… I see myself as a part of the wider medical community. I did not feel that at all during pre-clerkship.”* (P6)

### Heroism

The pandemic evoked a profound sense of heroism and heightened appreciation for the invaluable services rendered by healthcare professionals. Mainstream media and society swiftly acknowledged the sacrifices made by healthcare professionals – risking their own well-being in service to the community. This lens celebrated healthcare professionals’ steadfast dedication, selflessness, and sacrifices in delivering care.

Among participants, the shift in societal perception impacted their PIF. There was a heightened awareness of their roles as medical learners and future healthcare providers refracted through a heroic perspective. One student eloquently captured this sentiment, reflecting, “Amidst COVID, we were deemed essential. It served as a constant reminder that our work holds significance (P5).” Another participant echoed this, recognizing the personal risks and sacrifices involved in providing care, noting, “While working amidst COVID, seeing others staying home made me realize the value of what we do (P4).” The phrase “healthcare hero” proliferated across social media platforms. A participant reflected on this label, remarking, “When I entered medical school, I didn’t perceive myself as a hero. I never considered putting myself in harm’s way… now, it’s an expectation [while working during COVID] (P17).”

### Delayed Transition to Clerkship: The late blooming effect

Due to COVID-19, changes to preclerkship, such as online learning and decreased exposure to the clinical environment, not only impacted academic responsibility, but also created gaps in knowledge acquisition and application. Participants remarked feeling uncomfortable with physical exams. For example, the foundational physical exam skills such as the cardiology and respiratory exams were initially taught online using pillows, creating a skills gap when practicing on real patients. Others noted *“standardized patients are healthy* (P 9)*,”* and it’s hard to know what “real” (sick) patients look like without exposure to the clinical environment. In contrast, during pre-clerkship in a pre-pandemic context, observations created opportunities to see ill patients and pathology building clinical acumen. This deprivation of authentic clinical exposure underscores a sociocultural gap in understanding disease presentations and patient care. One participant highlighted this gap by explaining the difficulty in treating an uncomplicated pneumonia during clerkship, citing that “*I read about pneumonia and how to treat it, but I didn’t go into a hospital and see a patient with pneumonia… it made this encounter more difficult* (P 6).” It was evident that there was delayed socialization in the pre-clerkship stage of training.

However, the disruption caused by COVID-19 led to a delay in this progression. Clerkship emerged as a pivotal phase where medical students swiftly acquired knowledge through direct exposure, bridging gaps and compensating for the delayed socialization experienced in pre-clerkship. This accelerated process differed significantly from the usual pace of socialization within UGME. For many students, this created a drastic transition from pre-clerk to clerk. One student describes the growing pains associated with that transition to clerkship:

*“In the first two months of clerkship, I still felt very scared, very anxious, and didn’t know what was going on, which I think happens to everybody even pre COVID and across all medical schools. But I think COVID intensified that because I had taught myself a lot of stuff. I was still relearning physical exams. I was not really aware of how the hospital worked because I hadn’t really done many clinical experiences. So I think COVID really widened that gulf of transitioning.”* (P20)

As expected, clerkship addressed these concerns. By engaging in patient care, embedding in the healthcare system, students began to feel comfortable with their clinical skills and management approaches. Participants acknowledged that they *“learned more through experiencing instead of through studying* (P12)*.”* From a sociocultural lens, learning through exposure in clerkship served to rectify the delayed socialization in pre-clerkship.

### Shifting Paradigms: Ungelling and restructuring of the clinical environment

Despite participants’ negative reflections on COVID-19’s impact on the UGME experience, some students noted a positive change in the culture of medicine related to personal health and wellness. Students expressed that training during COVID-19 meant that *“if you were sick, you’re sick* (P18)*,”* and you were not expected to come in. Participants were aware that this was a drastic change from pre-pandemic perceptions of illness where you *“just go to work [sick] and suck it up* (P18)*.”* Others remarked that this sentiment was a cultural norm held for decades in the medical profession, strongly rooted in its identity, commenting that regardless of personal circumstances there were *“no days off whatsoever* (P10)*.”* One student explained that COVID changed the healthcare professional’s perception of illness, putting at the forefront their own personal health:

*“If a patient was code bluing and needed to be intubated, you used to just run in, forget everything else, run and intubate. ‘Cause it’s an emergency, they’re dying.’ But with COVID, I was told that now, even if it was an emergency and they’re code blue and they need intubation, you would still take that extra couple of seconds to put on a gown and then go in. That is an act to me that’s symbolic of the fact that you are putting your own mask on in an airplane first before you put on someone else’s mask.”* (P17)

Wellness and burnout were also at the forefront of interview discussions. Participants were more aware of healthcare professional burnout noting that *“with the pandemic you notice it [burnout] a lot more. People talk about it more and you witness it in your colleagues* (P9)*.”* Others were quick to recognize that “*burnout is not a product of COVID, it’s been drastically exacerbated by COVID* (P15)*.”* Through participating in conversations about, witnessing, and potentially experiencing burnout, participants had a perception that *“the pandemic shifted thinking into the fact that healthcare workers still deserve to have reasonable qualities of lives* (P11)*.”* This perceived sociocultural change in which physician wellness was more openly discussed, motivated dialogue among participants on prioritizing self-care and boundary setting. Our participants recognized the *“the importance of finding space between yourself and your work* (P6)*.”* They also commented on the *“need to have clear physical and emotional and mental boundaries for yourself* [and recognizing] *what you can provide and what you can’t* (P17)*.”*

During the COVID-19 pandemic, a perceived sociocultural shift unfolded within healthcare spaces. It encompassed alterations in illness policy, heightened acknowledgment of burnout, and swift restructuring of clinical settings. This pace of change cultivated a perception among participants that the profession was malleable across various domains as compared to the pre-COVID era. For example, several participants commented on the challenges of clerkship and the unexpected hurdles of procuring time off. Many expected flexibility with time off expecting wellness days, opportunities rarely provided for healthcare staff. One participant commented that “*more scheduled breaks or at least like one or two wellness days [are required]* (P10)”. Another echoed this sentiment questioning why “*we can’t get even one day off (*P15).” Participants perceived that the culture of medicine in relation to wellness and illness had shifted drastically. They noted a heightened empathy toward prioritizing personal well-being, which led to perceptions of a more adaptable professional landscape within other aspects of PIF.

### COVID-19’s Influence on PIF: The Ultimate Tally

When participants were prompted to reflect on the influence of the COVID-19 pandemic they strongly asserted that these shifts in perception would have taken place regardless of COVID-19. When directly questioned about whether the pandemic affected their PIF, 16 out of 20 participants responded no, indicating they did not perceive any influence of COVID-19 on their PIF in UGME.

One participant commented that *“most challenges as a medical student in COVID were probably similar to a medical student before COVID* (P13)*.”* Others agreed that the pandemic did not create disorienting dilemmas, rather the *“pandemic changed their relationship with the profession and what it meant to be a healthcare provider* (P4)*.”* Medical students were adamant that *“COVID-19 didn’t build character, it revealed it* (P7)*.”* The challenges faced by this particular cohort are deeply rooted in their unique medical school experience. They have only encountered one educational setting, never having the opportunity to compare a predominantly virtual curriculum to an in-person predominant one or gain insights from faculty or peers who have experienced both. Participants were aware of this challenge. One commented that “*it’s hard to compare medical school experience .. I never got clerkships before the pandemic to be able to compare to* (P18).” Another added a similar sentiment explaining “*I don’t have anything to compare it to. I don’t know if med school would have been like this or different* (P15).”

### Discussion

Our study delves into the impact of the COVID-19 pandemic on PIF in UGME from a sociocultural context. Abrupt shifts to online learning, increased social isolation, and limited in-person opportunities produced a disruptive environment. Medical students felt disconnected from their peers, educators, and the clinical environment. As they progressed into clerkship, they became aware of gaps in knowledge acquisition and application. Additionally, the pandemic heightened students’ awareness on cultural shifts in medicine particularly related to self-care and illness, leading to discussions regarding wellness and change cadence. Interestingly despite these observations, student participants perceived no change in their PIF trajectory due to the COVID-19 pandemic.

The COVID-19 pandemic was a sociocultural factor influencing the PIF of medical students, reshaping their attitudes, perceptions, and behaviors. The pandemic highlighted how social, cultural, and political dynamics due to changes in the environment impact students’ interactions within the healthcare system and their perceptions of healthcare professionals [4,14]. Despite these profound shifts we determined that while PIF was delayed, it was ultimately preserved within this cohort of medical students. This indicates that amid sociocultural shifts, PIF is an adaptable process. We can leverage this insight to explore effective ways of nurturing PIF based on our environment. This proactive approach enhances preparedness for future disruptive scenarios [18].

### Role Models and Experiential Learning: Fostering Growth and Development

This study highlights the importance of role models shaping PIF in pre-clerkship. In our study, we observed that social isolation resulting from the pandemic led to reduced interactions between students and their role models/mentors during pre-clerkship. This temporary separation and subsequent reunion provided additional evidence on the significance of these professional relationships [7,26,27]. While others have found professional socialization to be continued in the absence of face-to-face interaction, we found the opposite [14,18]. From a socialized context, McMaster University’s approach to education revolves around problem-based learning (PBL) and places a strong emphasis on team building. Within a socialized UGME context, online methods are notably deficient. Moreover, separation from the clinical environment emerged as a significant obstacle to PIF. Existing research suggests that hands-on experience in patient care plays a crucial role in fostering engagement with healthcare delivery [3,10]. Our findings strongly support this notion. We observed a challenging transition for study participants as they moved from preclerkship to clerkship. Limited opportunities for engaging in patient care during preclerkship left students with little awareness of their roles and responsibilities as clerks [4,26].

### Exploring Value-Taking: Unveiling Impacts and Examining Implications

#### Evaluating Physician Wellness In PIF Processes

Throughout the pandemic, students keenly observed a rise in burnout among peers and educators [28,29]. At the start of COVID-19 pandemic, students began their medical journey in a secluded Zoom environment, disconnected from robust sociocultural influences [26]. Moving to the more dynamic clerkship phase, students observed healthcare professionals grappling with burnout and actively engaged in numerous conversations about wellness alongside their role models and mentors. This led them to prioritize wellness in their PIF, highlighting the pivotal role of personhood in PIF [30].

This illustrates how the evolving social values within the profession catalyze shifts in the sociocultural elements of PIF. Sociocultural elements, such as exposure to real-world experiences and interactions with the medical community (e.g. engaging in conversations about wellness alongside mentors/role models) significantly influence the assimilation of specific values that shape professional identity. The COVID-19 pandemic, as a sociocultural factor, ushered in an environment where the delicate interplay between experiences, societal influences, and professional identity development, took on a dynamic nature [14,15]. It created space for dialogue on the broader values of the medical profession. Thus, we came to learn about personhood as a foundational element of PIF. We can observe this perception influencing organizational change, particularly in the evolution of the CanMEDS competency framework. CanMEDS delineates the essential skills physicians need to adeptly address the healthcare needs of their communities. These skills are categorized into seven roles: medical expertise, communication, leadership, advocacy, scholarship, and professionalism. Notably, the 2025 CanMeds working group has placed a significant emphasis on the emerging theme of physician humanism within this framework [31].

#### Change Cadence

The healthcare environment underwent a significant overhaul during the pandemic, such as shifts in perception of illness and restructuring of clinical settings. This rapid evolution instilled among student participants an expectation of flexibility. This created skewed expectations of the clinical environment. Students strongly voiced a desire for wellness days and scheduled breaks, elements typically absent within healthcare settings. Sociocultural theory underscores how individuals’ cognitive and identity development intertwines with societal contexts. Amid the pandemic, participants proactively adjusted their expectations of PIF surrounding cultural norms on wellness. This reshaped participant’s perceptions of professional identity, fostering an anticipation of adaptability in PIF post-pandemic. While literature specifically outlining the impacts of these expectations remains limited, tangible effects have emerged. There has been an observable surge in policy changes within residency programs. For example, the Professional Association of Residents of Ontario, spearheaded numerous reforms for residents amid the pandemic including guidelines on call scheduling, weekends off, and augmenting stipends [32]. The influence of the environment on shaping expectations was similarly observed with the Okanagan Charter, a framework urging institutions to prioritize student well-being. As this notion permeated medical education, it generated an expectation for medical schools to reform the learning environment to be ‘health-promoting’ and address issues within the culture of medical education.

#### Heroism

During the pandemic, the emphasis on service and the self-sacrifice of healthcare professionals spread within mainstream media. This portrayal of healthcare professionals as ‘heroes’ in social perception became a professional value adopted by study participants in their PIF, imbuing their work with purpose. It illustrates the influence of social attitudes and cultural perceptions on the adoption of values within the profession. The literature documents the “healthcare hero” phenomenon highlighting how portraying healthcare workers as heroes heightened a sense of duty and commitment [33,34,35]. For medical students, this also affected self-perception of their role on a healthcare team [36,37]. Cultural and societal perceptions of healthcare professionals can shape individuals’ perception and adoption of core values and beliefs central to PIF. Reiterating the influence of our environment and its intersection with PIF. In this instance, we witness the profound influence of cultural perceptions of healthcare, notably the depiction of healthcare workers as heroes, shaping participants’ perspectives on their professional identity.

### COVID-19 and PIF: Perceptions

In light of these shifts in PIF, characterized by both the adoption of new and heightened emphasis on existing values, it is perplexing how this cohort of medical students perceived no substantial change in their PIF as a result of the COVID-19 pandemic. It can be posited that the pandemic created a blind spot. Medical students during this time lacked the typical ‘mirror’ relationship with their colleagues that exists in a non-pandemic context, making the changes less apparent and harder to discern. A ‘mirror’ relationship is a process where learners can compare experiences and behaviors with those of their peers to gain insight on personal development [38,39]. It is a longitudinal experience that serves as a reflective tool to evaluate one’s professional growth. With social isolation during the pandemic, it is posited that the environment was not conducive to this reflection, thus impairing students’ ability to recognize shifts in their PIF.

Ultimately, despite initial hurdles our study demonstrates the resilient and adaptable nature of PIF. It highlights the capacity of medical students to navigate challenges and evolve in their professional development [12]. PIF is a dynamic process that can withstand and adjust to various circumstances, including disruptive environments.

### Future Directions

Our study emphasizes that the development of professional identity is not solitary; instead, students must navigate their evolving PIF within the rich sociocultural fabric that surrounds them. Therefore, it is crucial for future research in UGME to explore how PIF is impacted in various disruptive environments beyond the COVID-19 pandemic [18,40]. Exploring if similar trends in PIF emerge in different disruptive contexts would provide valuable insights into the theoretical foundations and domains underlying PIF. In addition, there is value in investigating the long-term effects of the COVID-19 pandemic on medical students’ perceptions of the clinical environment, their roles as a learner, and their transition to residency. It would be beneficial to explore these effects from various perspectives, including that of educators and interprofessional colleagues [18]. By understanding how the pandemic has influenced students’ attitudes and expectations, we can gain insights that inform future approaches to medical education and help shape supportive strategies.

The incorporation of personhood as a component of PIF requires additional investigation. It is important to explore the extent and manner in which personhood should be emphasized, considering its implications for medical school structure and content delivery. Additionally, understanding the consequences of the incorporation of personhood on learners’ PIF is essential for shaping educational practices. By exploring these areas, future research can enhance our understanding of PIF, inform educational practices, and promote a more inclusive and equitable approach to professional identity development in the face of disruption.

### Limitations

Our findings represent the impact of COVID-19 on PIF in a highly socialized PBL-based UGME program. COVID may have impacted PIF in medical students in less socialized, didactic focused curricula differently. We therefore invite future studies to include schools with different curricular structures, or expanding to include perspectives of a broader stakeholder group, such as educators. We focused on the experiences of two cohorts of medical learners from the class of 2022 and 2023. The class of 2022 had already transitioned to residency. As a result, the insights they offered may have been influenced by their experiences as junior medical residents.

### Conclusion

In summary, the COVID-19 pandemic triggered substantial sociocultural shifts in the UGME experience. The shift to online learning led to social isolation and postponed face-to-face patient interaction, altering medical students’ perceptions of the clinical environment. This period of disruption introduced new sociocultural influences shaping learners’ PIF, stimulating discussions about change agency, the significance of personhood, and the adoption of new values like wellness and heroism.

Despite these changes, our study illustrates that PIF is an adaptable process, even amidst disruptive environments like the COVID-19 pandemic. It showcases the capacity of learners to navigate and uphold their professional identity despite environmental change. Future research must prioritize a sociocultural perspective to fully comprehend the dynamics of PIF in disruptive contexts.

## Additional File

The additional file for this article can be found as follows:

10.5334/pme.1308.s1 Supplementary Material. Appendix A–Appendix C.
