# Supplementary material for: Quarantining From Professional Identity: How Did COVID-19 Impact Professional Identity Formation in Undergraduate Medical Education?
Source: Perspect Med Educ. 2024 Feb 20;13(1):130–40. doi: 10.5334/pme.1308 (PMC10885846; doi:10.5334/pme.1308)
Supplement: Supplementary Material. — Appendix A–Appendix C. [file pme-13-1-1308-s1.pdf]

Appendix A

INTERVIEW GUIDE

Part A: Interviewee introduction and setting the interview

Q1. Please tell me about yourself. (interviewer introduce themselves first)

Possible probes

- What year of medicine are you currently in?
- Are you living in Hamilton or commuting?
- Are you a first-generation medical student?

Q1. How did you come to medical school? Is this something you always suspected?

Q2. Please tell me about your experience in the undergraduate medical program and how you believe it has been impacted by COVID-19?

Possible probes

- Were any months that were particularly challenging?
- How did you find the transition to medical school?
- What complexities did you find in terms of medical school in the covid era ?
- How were your educational experiences before medical school?

Q3. What clinical learning and socialization opportunities have you been exposed to so far in UGME?

Possible probes

- Have you had to organize any events yourself? Tell me about it.
- How has the structure of these opportunities been impacted? (For example, doing learning or socialization activities online instead of in person). Has it made your medical education experience better or worse? Why?
- How confident do you feel in your clinical skills? Do you think this has been impacted by Covid restrictions?
- What has not changed in regard to your learning, socialization, or opportunities due to COVID-19?

Part B: Professional Identity Formation

Q4. How satisfied are you with your undergraduate medical experience (thus far)?

(1= not satisfied to 5 = extremely satisfied)

- What has contributed to this?

Q5. Did you have expectations for medical school in COVID? If so what where these expectations and where they me?

a. Do you find yourself comparing your journey to medical students at other universities?

Q6. Professional Identity Formation is defined as the process of internalizing a profession's core values and beliefs and is an explicit goal of undergraduate medical education.

a. How has your (professional) identity shifted during your medical career? What core values and beliefs have you internalized?
b. How do you think your Professional Identity Formation has been impacted by COVID?
c. What experiences have been pivotal in shaping your professional identity formation (during your undergraduate medical education)?

Possible probes

- How do you compare your professional identity (formation) to others in your class?

Q7. The TIME framework is a framework created by the University of Texas to outline humanities and social science-based competencies in medical education, specifically focusing on professional identity development. The framework highlights 6 domains of Professional Identity Formation including attitudes, personal characteristics, duties and responsibilities, habits, relationships, perception and recognition.

- Explain what each one of them means (ppt slide)

a. In your opinion, which of these domains has been impacted the most and why?
b. In your opinion, which of these domains has been impacted the least and why?
c. How have you and the medical school mitigated any impacts on these domains?

Q8. How has your professional(?) socialization changed since starting medical school?

a. What changes made during COVID would you like to keep and what changes should be reverted once the pandemic is over? Why?

Q9. Is there anything else you would like to talk about regarding your professional identity formation experiences in medical school?

Appendix B

Coding Framework

Theme	Categories	Examples
**Relationship Building and Connection**	Interpersonal	Developing meaningful connections with classmates. Sense of collegiality/community. 'Feeling like a medical student.'
	Systemic / Socialization	Sense of belonging in the medical community. 'Feeling part of the healthcare team.'
	Role Modeling	Who is considered a role model? How are they chosen? How do they influence PIF?
**Personal Characteristics**	Critical Thinking and Self-Awareness	Embodying reflexivity and critical consciousness. For example, not repeating mistakes and understanding gaps in performance/knowledge.
	Resilience and Adaptability	Being able to encounter challenges and respond from a growth mindset perspective. Where they are able to adapt to COVID changes (i.e., online learning).
	Self-Care	Mental health and medical school. Recognize components of burnout. Preventative and responsive measures.
	Expectations	What were the expectations coming into medical school? How did this impact perception of experience?
	Emotional Intelligence	Process of learning the nuances of professional-patient relationships. For example: balancing empathy with professionalism.

**Facets of Career Development**	Duties and Responsibilities / Transition To Residency	Ability to recognize the roles and responsibilities of a healthcare professional. For example, patient care, paperwork, confidentiality, disclosure. Difference between expectation vs. reality.
	Medical Knowledge Enhancement	How virtual learning and associated changes impacted knowledge enhancement.
	Patient-Centeredness	From objectification to empowerment of the patient. How patient care influenced identity development.
	Systems Thinking	From focusing on individual patients to understanding and utilizing the health system.
	Understanding Diversity	From seeing people as the same to recognizing uniqueness and individual differences.
**Components of Transformation**	Disorienting Dilemmas	Experience during medical training that triggers reflection, challenges previous assumptions, facilitates growth.
	Critical Reflection	Recognition of the disorienting dilemma coupled with assessment of assumptions and feelings.
	Discourse	Identifying and understanding new values, roles, or relationships.
	Action	Acquisition of knowledge and skills. Integration of new perspectives.

APPENDIX C

UGME Sample Schedule

<table>
<tr><th>PRECLERKHIP</th><td colspan="2">Medical Foundation One</td><td colspan="2">Medical Foundation Two</td><td colspan="2">Medical Foundation Three</td><td colspan="2">Medical Foundation Four</td><td colspan="2">Integration Foundation</td><td colspan="2">Pre-Clerkship Electives</td><td colspan="2">Transition to Clerkship</td></tr>
<tr><th>CLERKHIP</th><td>OB/GYN</td><td>EL</td><td>Psych</td><td>EL</td><td>FM</td><td>Ped</td><td>EL</td><td>ER</td><td>Surg</td><td>EL</td><td>Med</td><td>Anes</td><td>Ortho</td><td>EL</td></tr>
</table>
